# Neuroprotective Activities of Palmitoylethanolamide in an Animal Model of Parkinson's Disease

**DOI:** 10.1371/journal.pone.0041880

**Published:** 2012-08-17

**Authors:** Emanuela Esposito, Daniela Impellizzeri, Emanuela Mazzon, Irene Paterniti, Salvatore Cuzzocrea

**Affiliations:** 1 Department of Clinical and Experimental Medicine and Pharmacology, School of Medicine, University of Messina, Messina, Italy; 2 IRCCS Centro Neurolesi “Bonino-Pulejo", Messina, Italy; University of Nebraska Medical Center, United States of America

## Abstract

The biochemical and cellular changes that occur following treatment with 1-methyl-4-phenyl-1,2,3,6-tetrahyropyridine (MPTP) are remarkably similar to that seen in idiopathic Parkinson's disease (PD). PD is characterized by the degeneration of dopaminergic nigrostriatal neurons, which results in disabling motor disturbances. Activation of glial cells and the consequent neuroinflammatory response is increasingly recognized as a prominent neuropathological feature of PD. There is currently no effective disease-modifying therapy. Targeting the signaling pathways in glial cells responsible for neuroinflammation represents a promising new therapeutic approach designed to preserve remaining neurons in PD. Chronic treatment with palmitoylethanolamide (PEA, 10 mg/kg, i.p.), initiated 24 hr after MPTP injection (20 mg/kg), protected against MPTP-induced loss of tyrosine hydroxylase positive neurons in the substantia nigra pars compacta. Treatment with PEA reduced MPTP-induced microglial activation, the number of GFAP-positive astrocytes and S100β overexpression, and protected against the alterations of microtubule-associated protein 2a,b-, dopamine transporter-, nNOS- positive cells in the substantia nigra. Furthermore, chronic PEA reversed MPTP-associated motor deficits, as revealed by the analysis of forepaw step width and percentage of faults. Genetic ablation of peroxisome proliferator activated receptor (PPAR)-α in PPAR-αKO mice exacerbated MPTP systemic toxicity, while PEA-induced neuroprotection seemed be partially PPARα-dependent. The effects of PEA on molecules typically involved in apoptotic pathways were also analyzed. Our results indicate that PEA protects against MPTP-induced neurotoxicity and the ensuing functional deficits even when administered once the insult has been initiated.

## Introduction

Parkinson's disease (PD) is the second most common neurodegenerative disease [1] and is characterized by progressive loss of substantia nigra neurons accompanied by the occurrence of Lewy bodies. The cause of idiopathic PD is still unknown but aging, environmental factors, oxidative stress, neuroinflammation and genetic factors may be involved in the development of the disease [2], [3], [4]. Research into the pathogenesis of PD has been rapidly advancing as a result of the development of animal models which also permit the investigation of new treatments.

Neurotoxin 1-methyl-4-phenyl-1,2,3,6-tetrahydropyridine (MPTP) animal model is a useful model for the study of neurodegeneration in Parkinson's disease because it produces clinical, biochemical and neuropathological changes similar to those observed in human PD [5]. MPTP was discovered in 1982, when a group of drug addicts developed subacute severe parkinsonism [6]. The neurotoxic effects of MPTP are thought to be mediated by its metabolite 1-methyl-4-phenylpyridinium ion (MPP+) which is caused by the oxidation of MPTP by monoamine oxidase-B (MAO-B) in glial cells [7]. Several cell death mechanisms involved in the neurotoxicity of MPTP have been suggested, including the inhibition of complex I in the mitochondrial electron transport chain, inflammation and the generation of reactive oxygen species, among others [5]. MPP+ is selectively taken up by the high affinity dopamine uptake system leading to a number of deleterious effects on cellular function, such as impaired intracellular calcium buffering, generation of free radicals from mitochondria and activation of neuronal nitric oxide synthase (nNOS), a calmodulin-dependent enzymes [8].

Moreover, in the last decade, evidence has accumulated of a central role of chronic neuroinflammation in PD pathogenesis, suggesting that anti-inflammatory treatments may represent a viable strategy in PD [9], [10], [11], [12].

PPAR agonists have also been assessed in a model of PD. In particular, oral administration of the PPARγ agonist pioglitazone attenuated the MPTP-induced glial activation and prevented dopaminergic cell loss in the substantia nigra pars compacta. Pioglitazone also prevented MPTP-induced expression of inducible nitric oxide synthase [13]. Preliminary results demonstrate that PPAR-alpha activation by fenofibrate prevents death of dopaminergic neurons of substantia nigra pars compacta in the MPTP model of Parkinson's disease, while another PPAR-α agonists bezafibrate had no effect in MPTP model. For this reason we used PPAR-αKO mice in the same model [14]. PPAR-α is known to be expressed by dopamine neurons of the substantia nigra and spiny neurons of dorsal striatum [15], [16], [17], [18], and might play antiinflammatory and antioxidant roles in the nigrostriatal circuit. PPAR-α has also been linked to activity of mesencephalic dopamine neurons, because activation of PPAR-α decreases *in vitro* both dopamine cell activity and ventral tegmental area net output [19]. Moreover, primary and cultured astrocytes express PPAR-α, as reported by Mattace Raso [20].

PPAR-α is a receptor for a diverse set of fatty-acid derivatives, including oleoylethanolamide – which binds to the purified ligand-binding domain of PPAR-α with a K_D_ of 40 nM and activates it with a median effective concentration (EC50) of 120 nM [21] – and palmitoylethanolamide (PEA), a compound whose profound anti-inflammatory effects are mediated by this receptor [22]. PEA was identified more than five decades ago [23] and was shown to reduce allergic reactions and inflammation in animals along with influenza symptoms in humans [24]. Moreover, PEA has been shown to inhibit peripheral inflammation and mast-cell degranulation [25], as well as to exert neuroprotective [26], [27], [28] and antinociceptive [29] effects in rats and mice. These actions are mediated by PPAR-α activation and are accompanied by a decrease in NO production, neutrophil influx [27], and expression of proinflammatory proteins such as inducible nitric oxide synthase (iNOS) and cyclooxygenase-2 (COX-2) [30].

Based on this evidence, in the present study, we assessed the neuroprotective effect of PEA and its relevance to PD sintomatology in the MPTP mouse model of PD. A comparison of PEA against minocycline, the currently approved treatment, was also performed.

## Methods

### Animals

Mice (6–7 weeks old, 20–27 g) with a targeted disruption of the PPAR-α gene (PPAR-αKO) and littermate wild-type controls (PPAR-αWT) were purchased from Jackson Laboratories (Harlan Nossan, Italy). Mice homozygous for the *Ppara^tm1Gonz^* targeted mutation (Strain Name: 129S4/SvJae-*Ppara^tm1Gonz^*/J) are viable, fertile and appear normal in appearance and behavior [31]. The study was approved by the University of Messina Animal Care Review Board. The animals were housed in a controlled environment and provided with standard rodent chow and water. Animal care was in compliance with regulations in Italy (D.M. 116192), Europe (O.J. of E.C. L 358/1 12/18/1986), and USA (Animal Welfare Assurance No A5594-01, Department of Health and Human Services, USA).

### MPTP-induced PD and treatment

Eight-week-old male PPAR-αKO and PPAR-αWT mice were treated with either MPTP hydrochloride or saline. For MPTP intoxication, mice received four intraperitoneal injections of MPTP-HCl (20 mg/kg; Sigma, St. Louis, MO) in saline at 2 hr intervals in 1 day: the total dose per mouse is 80 mg/kg. For PEA treatment (10 mg/kg in 10% polyethylene glycol and 5% TWEEN 80 sterile distilled water, i.p. bolus) mice received intraperitoneal injections of PEA starting 24 hrs after the first MPTP injection and continuing through 7 additional days after the last injection of MPTP. Minocycline (50 mg/kg i.p.) was administered at 24 h after the first MPTP injection and this was followed 24 h later by a second injection of 50 mg/kg. Subsequently, treated mice were injected with a 25 mg/kg dose every 24 h for the next 6 days. Sham animals received vehicle only. Animals were killed 8 days after MPTP injection and their brains were harvested, sectioned, and processed [32]. PEA was purchased from Tocris Bioscience (UK); the doses of PEA (10 mg/kg) used here were based on previous in vivo study [27], [33]. Minocycline (Sigma, St Louis, MO, USA) dose is comparable with that used in the experimental literature [34], [35], [36]. Mice were randomly allocated into the different experimental groups (n = 30/each group). In the experiments regarding the behavior, the animals were ten/group.

### Immunohistochemical localization of TH, DAT, S100β, MAP-2, iNOS, and PAR

The mice were anesthetized with sodium pentobarbital (50 mg/kg, i.p.) 8 days after MPTP treatment, and the brains were perfusion-fixed with 4% paraformaldehyde in 0.1 M phosphate buffer (pH 7.4) following a heparinized saline flush. The brains were removed 1 h after perfusion fixation at 4°C and were immersed in the same fixative solution.

The brain sections were then dehydrated with graded ethanol, passed through chloroform, and embedded in paraffin. Paraffin sections, 5 µm, of the striatum and substantia nigra were used for immunohistochemistry.

After deparaffinization, endogenous peroxidase was quenched with 0.3% (v/v) hydrogen peroxide in 60% (v/v) methanol for 30 min. The sections were permeabilized with 0.1% (w/v) Triton X-100 in PBS for 20 min. Non-specific adsorption was minimized by incubating the section in 2% (v/v) normal goat serum in PBS for 20 min. Endogenous biotin or avidin binding sites were blocked by sequential incubation for 15 min with biotin and avidin (Vector), respectively. Sections were incubated overnight with anti-PAR(GeneTex, 1∶500 in PBS, v/v; catalog #GTX75054), anti-iNOS antibody (Santa Cruz Biotechnology, 1∶500 in PBS, v/v; catalog #sc-8310), anti-MAP-2 rabbit polyclonal antibody (Millipore, 1∶500 in PBS, v/v; catalog #AB5622), with anti-S100β antibody (Abcam,1∶500 in PBS, v/v; catalog #AB4066), anti-DAT antibody (Santa Cruz Biotechnology, 1∶500 in PBS, v/v; catalog #sc-14002), anti-TH polyclonal antibody (Santa Cruz Biotechnology, 1∶500 in PBS, v/v; catalog #sc-14007), or anti-PPAR-α rabbit polyclonal antibody (Santa Cruz Biotechnology, 1∶500 in PBS, v/v; catalog #sc-9000).

Sections were washed with PBS, and incubated with secondary antibody. Specific labeling was detected with a biotin-conjugated goat anti-rabbit IgG and avidin-biotin peroxidase complex (Vector Lab. Inc., Burlingame, CA). To verify the binding specificity for different antibodies, some sections were also incubated with only the primary antibody (no secondary) or with only the secondary antibody (no primary). In these situations no positive staining was found in the sections indicating that the immunoreactions were positive in all the experiments carried out. Immunocytochemistry photographs (n = 5 photos from each samples collected from all mice in each experimental group) were assessed by densitometry as previously described [37], [38] by using Optilab Graftek software on a Macintosh personal computer.

### Western blot analysis for iNOS, Bax, Bcl-2, GDNF, NGF, NT-3, and GFAP

Brain tissue was disrupted by homogenization on ice in lysis buffer (HEPES 20 mm, pH 7.9; 420 mm NaCl; 1.5 mm MgCl2; 1 mm EGTA; 0.2 mm EDTA; 25% (vol/vol) glycerol; 0.5% Nonidet P-40; 0.5 mm phenylmethylsulfonyl fluoride; 1.5 µg/ml trypsin inhibitor; 3 µg/ml pepstatin A; 2 µg/ml leupeptin; 0.1 mm benzamidine; and 0.5 mm dithiothreitol), as previously described [39]. After 1 h, tissue lysates were obtained by centrifugation at 100,000×g for 15 min at 4 C. Protein concentrations were estimated by the Bio-Rad protein assay (Bio-Rad Laboratories, Segrate, Milan, Italy) using BSA as standard. For Western blot analysis, 25 or 40 µg protein of lysates was dissolved in Laemmli's sample buffer, boiled for 5 min, and subjected to SDS-PAGE (8% and 15% polyacrylamide). The blot was performed by transferring proteins from a slab gel to nitrocellulose membrane at 240 mA for 40 min at room temperature. The filters were blocked with 1× PBS, 5% (w/v) non fat dried milk (PM) for 40 min at room temperature and subsequently probed with specific Abs anti-iNOS (BD Transduction Laboratories, 1∶500, 1∶1000; catalog #610432), anti-Bax (Santa Cruz Biotechnology, 1∶500; catalog #sc-526), or anti-Bcl-2 (Santa Cruz Biotechnology, 1∶500; catalog #sc-492), or anti-GDNF, (Santa Cruz Biotechnology, 1∶1000; catalog #sc-9010) or anti-NGF (Santa Cruz Biotechnology, 1∶1000; catalog #sc-549), anti-GFAP (Santa Cruz Biotechnology, 1∶500; catalog #sc-65343) or anti-NT-3 (Santa Cruz Biotechnology, 1∶1000; catalog #sc-547) in 1× PBS, 5% w/v non fat dried milk, 0.1% Tween-20 (PMT) at 4°C, overnight. Membranes were incubated with peroxidase-conjugated bovine anti-mouse IgG secondary antibody or peroxidase-conjugated goat anti-rabbit IgG (Jackson ImmunoResearch, West Grove, PA, 1∶2000) for 1 h at room temperature. Polyclonal anti-β-actin (Sigma, 1∶10000) was used as an internal standard for cytoplasm fractions.

The relative expression of the protein bands of iNOS (∼130 kDa), Bax (∼23 kDa), Bcl-2 (∼29 kDa), was quantified by densitometric scanning of the X-ray films with GS-700 Imaging Densitometer (GS-700, Bio-Rad Laboratories, Milan, Italy) and a computer program (Molecular Analyst, IBM).

### Nuclear protein extraction and PPAR-α expression by Western blot

Brain tissues were obtained from the animals of all experimental groups were homogenized on ice in ice-cold hypotonic *buffer A* (10 mM Hepes pH 7.9, 10 mM KCl, 0.1 mM EDTA, 0.1 mM EGTA, 1 mM DTT, 0.5 mM PMSF with a protease inhibitor cocktail) using a Politron PT 13,000 D tissue homogenizer (Kinematica). After 15 min incubation on ice, the homogenates were centrifuged at 1,000 g for 10 min at 4°C. Supernatants containing cytoplasm extracts were stored at −80°C. Nuclear pellets were resuspended in ice-cold *buffer B* (1% Triton X-100, 150 mM NaCl, 10 mM TRIS-HCl pH 7.4, 1 mM EGTA, 1 mM EDTA, 0,2 mM PMSF, 20 mm leupeptin, 0,2 mM sodium orthovanadate) and the tubes were vigorously rocked at 4°C for 30 min on a shaking platform. The nuclear extracts were centrifuged at 13,000 g for 15 min at 4°C. The supernatants were frozen in aliquots at −80°C until use. Proteins from nuclear fraction were added to sample buffer, and boiled in a water bath for 5 min. Protein samples (40 mg per lane) were separated on denaturing 10% SDS polyacrylamide gel and transferred to a nitrocellulose membrane. Non-specific binding to the membrane was blocked for 1 h at room temperature with 5% milk in PBS. Membranes were then incubated at 4°C overnight with primary antibody in milk-PBS- Tween 20 0.1% (PMT) for PPAR-α, (Santa Cruz Biotechnology, 1∶500; catalog #sc-9000), washed three times with PBS-0.1% Tween, and then incubated for 1 h at room temperature with a secondary antibody (peroxidase-conjugated goat anti-rabbit IgG, 1∶2000; Jackson ImmunoResearch, West Grove, PA). Polyclonal anti-lamin A/C antibody (Sigma, 1∶5000;) was used as an internal standard for nuclear fractions. The immunoreactive bands were visualized using an enhanced chemilumunescence system (SuperSignal West Femto Maximum Sensitivity Substrate, Pierce). The protein bands were scanned and quantitated with Gel Doc-2000 (Bio-Rad).

### Light microscopy

The mice were anesthetized with sodium pentobarbital (50 mg/kg, i.p.) 8 days after MPTP injection, and the brains were perfusion-fixed with 4% paraformaldehyde in 0.1 M phosphate buffer (pH 7.4) following a heparinized saline flush. The brains were removed 1 h after perfusion fixation at 4°C and were immersed in the same fixative solution. The brain sections were performed for 24 h in the same solution. The brain sections were then dehydrated with graded ethanol, passed through chloroform, and embedded in paraffin. Paraffin sections, 5 µm, of the striatum and substantia nigra were used for histological coloration. Tissue sections were deparaffinized with xylene, stained with Haematoxylin/Eosin (H&E) and studied using light microscopy (Dialux 22 Leitz).

### Behavioral testing

Behavioral assessments on each mouse were made 1 day prior to, and 8 days after, MPTP injection. Motor performance was assessed with a rotary rod apparatus using a protocol similar to that described [40],[41]. For the rotarod tests the rotadrum was filled with water to a level just below the bottom of the rod. The mice were placed on the rotating rod and the time until they fell off was recorded. This was repeated (with a rest period that increased by 5 s with each fall) until the total time on the rod for the control group was 5 min. Both the total time spent on the rotating rod and the total number of falls for each mouse were recorded.

### Catalepsy test

Catalepsy, defined as a reduced ability to initiate movement and a failure to correct posture, was measured by means of the bar test. To test for catalepsy, mice were positioned so that their hindquarters were on the bench and their forelimbs rested on a 1 cm diameter horizontal bar, 4 cm above the bench. The length of time the mice maintained this position was recorded by stopwatch to a maximum of 180 s. The catalepsy test was evaluated at 8 days after MPTP injection [42]. Mice were judged to be cataleptic if they maintained this position for 30 s or more. Animals were put back in their home cage after each measurement of catalepsy.

All values are expressed as the means±SEM.

### Beam traversal test

Behavioral testing on the beam traversal test was carried out prior to treatment, and at 8-days of treatment prior to sacrifice as described [43]. In the beam traversal test, a measure of motor performance, mice were trained over two days, with 5 trials per day, to cross a narrowing beam (separated into four segments) with support ledges attached along each side, and leading to the animal's home cage. The test was made more challenging by placing a mesh grid over the beam surface, leaving a small space of about 1 cm between the grid and the surface of the beam. Animals were then videotaped over a period of 5 trials, and the time to cross, number of steps taken and number of slips were recorded by an investigator blind to drug treatment.

### Materials

All compounds were obtained from Sigma-Aldrich Company Ltd. (Milan, Italy). All other chemicals were of the highest commercial grade available. All stock solutions were prepared in non-pyrogenic saline (0.9% NaCl; Baxter, Italy, UK).

### Statistical evaluation

All values in the figures and text are expressed as mean ± standard error of the mean (SEM) of N observations. For the in vivo studies N represents the number of animals studied. In the experiments involving histology or immunohistochemistry, the figures shown are representative of at least three experiments (histological or immunohistochemistry coloration) performed on different experimental days on the tissue sections collected from all the animals in each group. The results were analyzed by one-way ANOVA followed by a Bonferroni post-hoc test for multiple comparisons. A p-value of less than 0.05 was considered significant. Basso-Beattie-Bresnahan (BBB) was analyzed by Mann-Whitney U statistics for comparisons between different data sets and considered significant when p- value was <0.05.

## Results

### PEA treatment reduced behavioural impairments induced by MPTP intoxication

#### Catalepsy test

**Figure 1:**
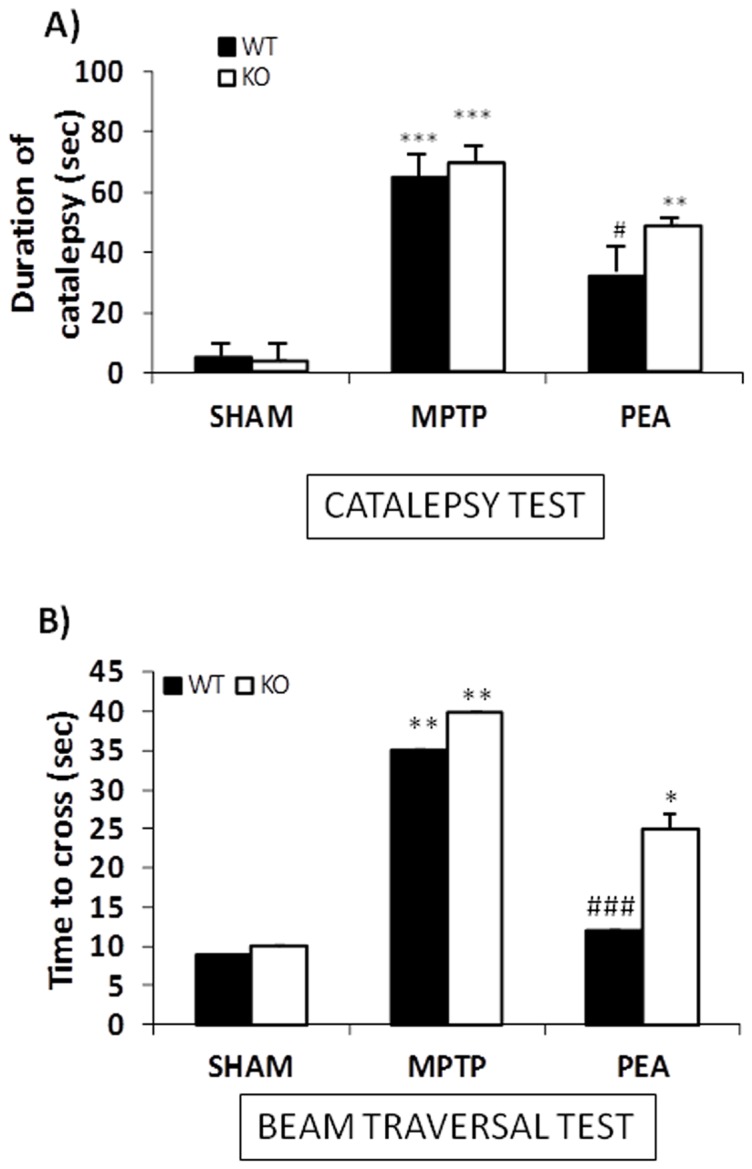
Effect of PEA on behavioural impairments induced by MPTP. Catalepsy was evaluated according to the standard bar hanging procedure (A). In panel B results from Beam Trasversal test are reported. PEA treatment (10 mg/kg) reduced behavioural impairments induced by MPTP in WT mice, but in PPARαKO mice PEA didn't ameliorate motor dysfunction induced by MPTP. *p<0.05, **p<0.01, ***p<0.001 vs Sham; #p<0.05, ##p<0.01 vs MPTP mice.

The MPTP administration produced a significant cataleptic response (Figure 1A). The duration of MPTP-induced catalepsy was significantly reduced by 10 mg/kg PEA.

The absence of PPAR-α gene significantly increases the motor deficits in catalepsy test. Treatment with PEA in PPAR-αKO mice did not reduced the motor abnormalities in the catalepsy test (Figure 1A). These results indicate that the PEA effect in PPAR-αKO mice was less marked than in WT thus suggesting that PPAR-α is important for the PEA-mediated effect.

#### Beam traversal test

PPAR-αWT and PPAR-αKO mice injected with MPTP and treated or not with PEA were tested for motor performance on the beam traversal test. A significant reduced motor performance was observed at 8 days after the MPTP as compared with vehicle (Figure 1B). MPTP injection in the PPAR-αKO mice induced a performance which was significantly worse as compared with WT, suggesting a endogenous protective role of PPAR-α in the regulation of the progression in neuropathology. PEA significantly (p<0.01) improved motor deficits only in PPAR-αWT following 7-days of treatment, PEA effect in PPAR-αKO mice was less marked than in WT thus suggesting that PPAR-α is important for the PEA effect (Figure 1B).

#### Rotarod test

**Figure 2:**
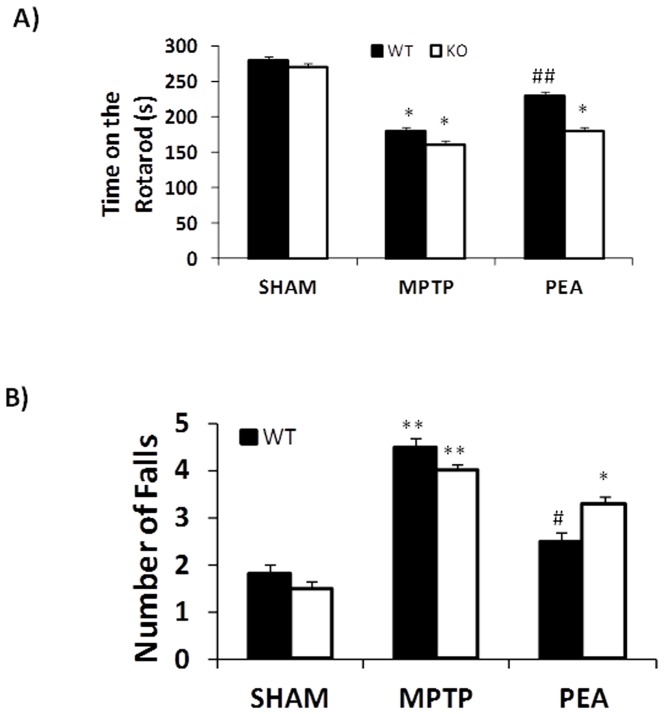
Effect of PEA treatment on motor function assessed by rotarod. Motor function after MPTP was also assessed using a rotarod apparatus. At 8 days after the MPTP injection, PPARαWT and PPARαKO mice exhibited a significant motor dysfunction as indicated by a decrease in the time period on rotarod and by an increased numbers of falls (A and B). The absence of PPARα gene significantly increases the motor dysfunction induced by MPTP. PEA treatment blunted the motor dysfunction in PPARαWT mice but not in PPARαKO mice.

Motor function of PPAR-αWT and PPAR-αKO mice after MPTP was also assessed using a rotarod apparatus. At 8 days after the MPTP injection, PPAR-αWT mice exhibited a significant motor dysfunction as indicated by a decrease in the time period on rotarod and by an increased numbers of falls (Figure 2A and B).

The absence of PPAR-α gene significantly increases the motor dysfunction induced by MPTP. The absence of PPAR-α gene alone did not impair performance on the rotarod tests. Treatment with PEA in PPAR-αWT mice but not in PPAR-αKO mice reduced the motor dysfunction induced by MPTP.

### PEA treatment reduced the loss of TH expression and plasma membrane dopamine transporter in the SN induced by MPTP administration

TH is the enzyme responsible for catalyzing the conversion of the amino acid L-tyrosine to dihydroxyphenylalanine (DOPA), that is a precursor for dopamine. As TH catalyses the formation of L-DOPA, Parkinson's disease can be considered as a TH-deficiency syndrome of the striatum.

**Figure 3:**
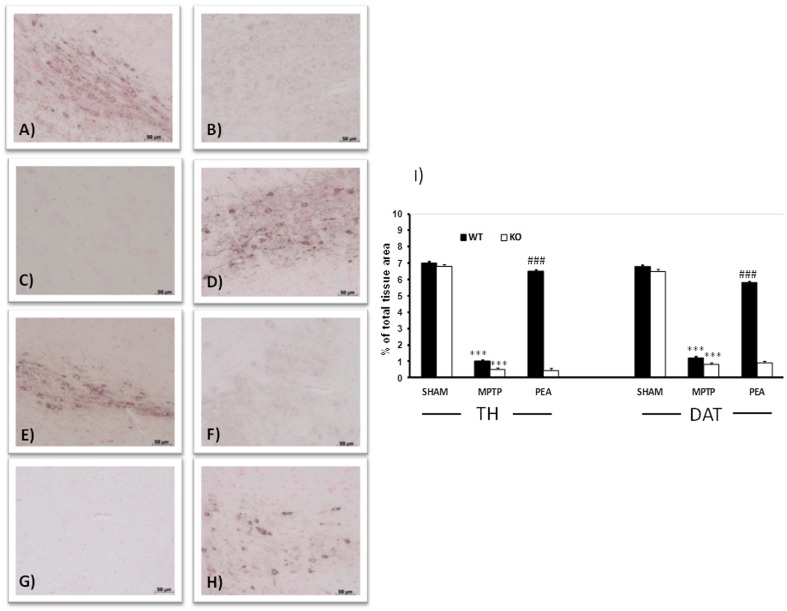
Effect of PEA on TH and DAT expression in SN. An important loss of TH-positive cells was observed in MPTP-injected animals at 8 days after intoxication in PPARαWT (**B, see** densitometric analysis in I). The absence of PPARα gene in PPARαKO significantly increases the loss of TH-positive cells after MPTP (C and I). The absence of PPARα gene alone did not modify the presence of TH-positive cells in sham animals (A). PEA reduced in PPARαWT mice the MPTP-induced loss of TH-positive neurons in the SN (D and I). Moreover, MPTP injection produced a strong loss of DAT expression at 8 days (F and I). The absence of PPARα gene increases the loss of DAT positive cells (G and I). In panel H and I, PEA treatment ameliorated in PPARαWT mice the sever reductions in levels of DAT. PEA failed to rescue DAT and TH induced by MPTP injection in PPAR-α KO mice.

In the SN, where the somata of dopaminergic neurons are located, an important loss of TH-positive cells was observed in MPTP-injected animals at 8 days after intoxication (Figure 3B). The absence of PPARα gene increases the loss of TH-positive cells induced by MPTP (Figure 3C and respective densitometric analysis). The absence of PPAR-α gene alone did not modify the presence of TH-positive cells in sham animals (Figure 3A). PEA prevented in PPAR-αWT mice the MPTP-induced loss of TH-positive neurons in the SN (Figure 3D and respective densitometric analysis) but PEA didn't prevent it in PPAR-αKO mice (see densitometric analysis). Similarly, in the striatum, the area of projection of dopaminergic neurons from the SN, a loss of TH immunoreactivity was observed in MPTP-treated animals at day 8 (data not shown). The absence of PPAR-α gene increases the loss of TH-positive terminals induced by MPTP also in the striatum (data not shown) and the treatment of PPAR-αWT mice but not of PPAR-αKO mice with PEA reduced the MPTP-induced loss of TH-positive terminals in the striatum (data not shown). Moreover, in order to better study the effect of PEA treatment on the dopamine pathway we have evaluated the levels of DAT, a member of a large family of Na^+^-Cl^−^ dependent transporters, which is thought to control the synaptic activity of released dopamine by rapid reuptake of the neurotransmitter into presynaptic terminals. MPTP injection produced an important loss of DAT expression at 8 days (Figure 3F and I). The absence of PPAR-α gene increases the loss of DAT positive cells induced by MPTP (Figure 3G and respective densitometric analysis). As shown in Figure 3H, PEA treatment ameliorated in PPAR-αWT mice the sever reductions in levels of DAT but not in PPAR-αKO mice (see densitometric analysis).

### PEA treatment reduced the alteration of S100β and MAP-2 expression in the SN induced by MPTP

**Figure 4:**
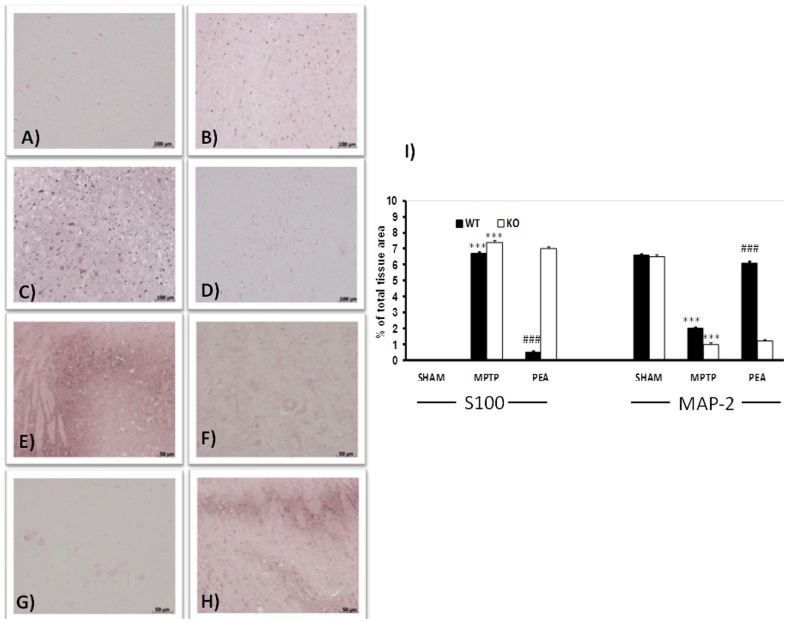
Effects of PEA on alteration of S100β and MAP-2 expression in the SN. A strong increase in S100β-immunoreactive cells was observed in MPTP-injected PPARαWT animals at 8 days (B and I). A stronger increase in number of S100β-immunoreactive cells was evident in PPARαKO (C and I). The absence of PPARα gene alone did not modify the presence of S100β-immunoreactive cells in sham animals (A). PEA reduced the increased expression of S100β-immunoreactive cells in the SN in PPARαWT mice (D and I). In addition, administration of MPTP to PPARαWT mice produced an important loss in MAP-2 expression at 8 days (F and I). The absence of PPARα gene significantly increased the loss of MAP-2 positive cells induced by MPTP (G and I). PEA treatment ameliorated in PPARαWT mice (H and I) the sever reductions in MAP-2 levels. PEA failed to rescue S100 and MAP-2 induced by MPTP injection in PPAR-α KO mice.

As showed in Figure 4B, an increase in S100β-immunoreactive cells was observed in MPTP-injected animals at 8 days. The absence of PPAR-α gene importantly increases the number of S100β-immunoreactive cells induced by MPTP (Figure 4C and respective densitometric analysis). The absence of PPAR-α gene alone did not modify the presence of S100β-immunoreactive cells in sham animals (Figure 4A). PEA prevented increased expression of S100β-immunoreactive cells in the SN in PPAR-αWT mice (Figure 4D and respective densitometric analysis) but not in PPAR-αKO mice (see densitometric analysis). Similarly, in the striatum an enhance in S100β-immunoreactive cells was observed in MPTP-treated animals at day 8 (data not shown). The absence of PPAR-α gene increases the number 0f S100β-immunoreactive cells induced by MPTP also in the striatum (data not shown). The treatment of PPAR-αWT mice but not of PPAR-αKO mice with PEA reduced the MPTP-induced increase of S100β-immunoreactive cells in the striatum (data not shown). In addition, we have also evaluate the effect of PEA treatment on MAP-2 expression. MAP-2 is a cytoskeleton protein mainly localized in neuronal dendrites and is known to stabilize microtubule assembly and mediate their interactions with other neuronal cell components. Administration of MPTP to mice produced a decrease in MAP-2 expression at 8 days (Figure 4F and densitometric analysis). The absence of PPAR-α gene increased the loss of MAP-2 positive cells induced by MPTP (Figure 4G and respective densitometric analysis). PEA treatment ameliorated in PPAR-αWT mice (Figure 4H and densitometric analysis) but not in PPAR-αKO mice (data not shown) the sever reductions in levels of MAP-2 expression.

### PEA treatment modulates the expression of iNOS in the SN induced by MPTP

**Figure 5:**
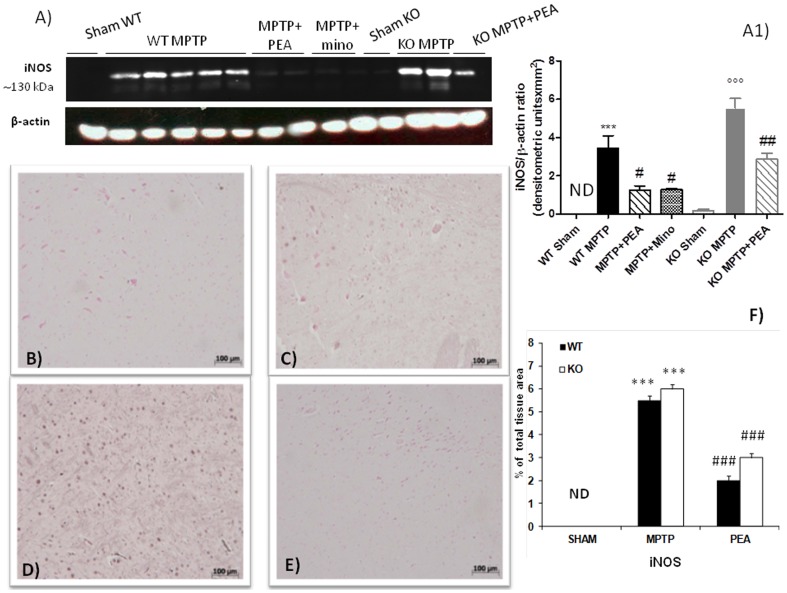
Effects of PEA on iNOS expression in the SN. By western blot analysis a significant increase in iNOS expression was observed in MPTP-injected PPARαWT mice (panels A and A1). In PPARαKO the increased iNOS expression was more pronounced. PEA treatment significantly lowered the expression of iNOS in PPARαWT mice and PEA partially reduced iNOS expression in PPARαKO mice (panel A and A1). Similarly, a marked positive immunostaining for iNOS was found in MPTP-injected PPARαWT animals at 8 days after intoxication (C and F). The absence of PPARα gene increases the number of iNOS immunoreactive cells induced by MPTP (D and F). In PPARαWT mice PEA prevented the MPTP-induced increased expression of iNOS immunoreactive cells in the SN (E and F). Sections from PPARαKO mice (B and F) did not reveal any immunoreactivity for iNOS.

To demonstrate the neuroinflammatory activity by which treatment with PEA may attenuate the development of PD, we also evaluated the expression of iNOS by western blot analysis and immunohistochemistry in the brain homogenates at 8 day after MPTP administration. By western blot analysis a significant increase in iNOS expression was observed in MPTP-injected PPAR-αWT mice (Figure 5, panels A and A1). The absence of PPARα gene significantly increases the expression of iNOS induced by MPTP. PEA treatment significantly reduced the expression of iNOS in PPAR-αWT mice and PEA still worked in PPAR-αKO mice (Figure 5, panel A and A1). In addition, a marked positive immunostaining for iNOS was found in MPTP-treated animals at 8 days after intoxication (Figure 5C). The absence of PPAR-α gene increased the number of iNOS immunoreactive cells induced by MPTP (Figure 5D). In PPAR-αWT mice PEA prevented the MPTP-induced increased expression of iNOS immunoreactive cells in the SN (Figure 5E). Sections of SN obtained from the sham PPAR-αWT mice (data not shown) and PPAR-αKO mice (Figure 5B) did not reveal any immunoreactivity for iNOS.

### Effects of PEA on PAR formation

**Figure 6:**
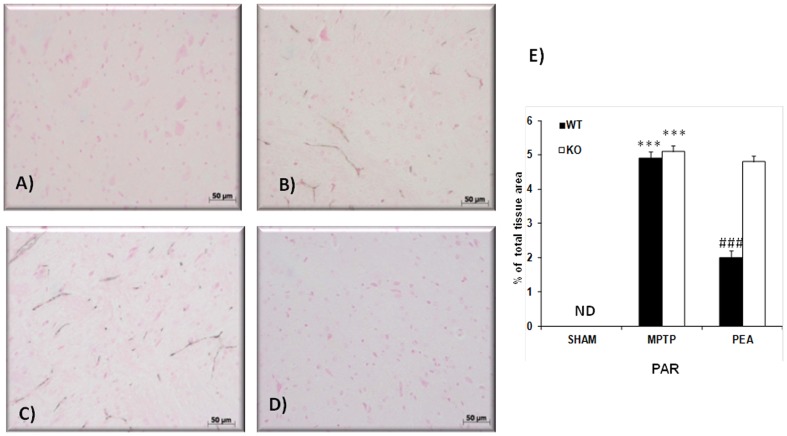
Effects of PEA on PARP activation. Brain sections were processed to determine the immunohistological staining for PAR, product of PARP activation. No positive staining for PAR observed in SN from PPARαKO sham-operated mice (A and E). A substantial PAR formation was found in inflammatory cells in SN from MPTP-injectd PPARαWWT and PPARαKO (B and C, respectively). PAR formation was significantly attenuated in PEA-treated MPTP PPARαWT mice (D and E). This figure is representative of at least 3 experiments performed on different experimental days.

SN sections from sham PPAR-αWT mice (data not shown) and PPAR-αKO mice (Figure 6A) did not stain for PAR, as an indicator of *in vivo* PARP activation related to DNA damage, whereas those obtained from PPAR-αWT mice at 8 day after MPTP intoxication exhibited positive staining for PAR (Figure 6B and E). The absence of PPAR-α gene importantly increased the number of PAR immunoreactive cells induced by MPTP (Figure 6C and E). PEA prevented in PPAR-αWT mice (Figure 6D and E) but not in PPAR-αKO mice (data not shown) the MPTP-induced increased expression of PAR immunoreactive cells in the SN.

### PEA treatment reduced the alteration of Bax and Bcl-2 expression in the SN induced by MPTP

**Figure 7:**
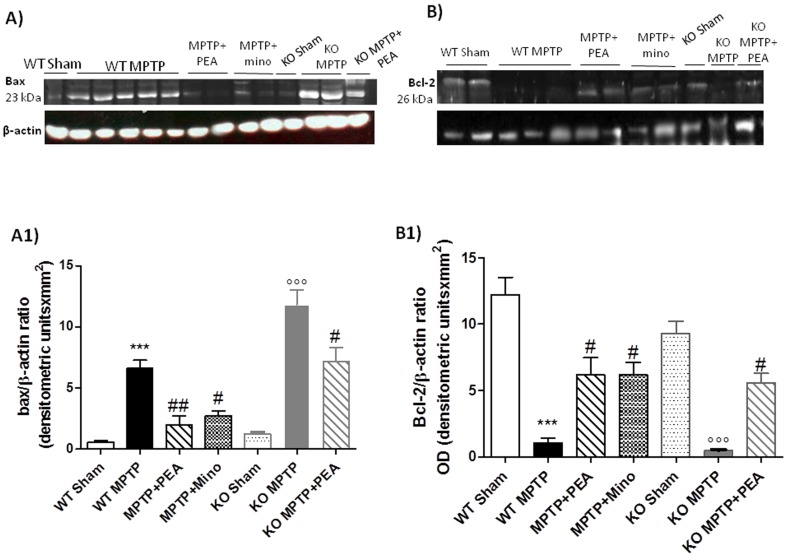
Effects of PEA on apoptosis. By Western blot analysis, Bax expression was appreciably increased in SN from PPARαWT and PPARαWT mice (panels A and A1) after intoxication. On the contrary, PEA treatment prevented the MPTP-induced Bax expression. Moreover, a basal level of Bcl-2 expression was detected in SN from PPARαKO sham-operated mice (panels B and B1). Eight days after MPTP, Bcl-2 expression was significantly reduced in SN from MPTP mice (panels B and B1). PEA significantly reduced the MPTP-induced inhibition of Bcl-2 expression (panels B and B1). The relative expression of the protein bands was standardized for densitometric analysis to β-actin levels, and reported in panel **a1** and **b1** are expressed as mean ± s.e.m. from n = 5/6 brains for each group. *P<0.01 *versus* sham, °*P*<0.01 *versus* MPTP+vehicle. No positive staining for Bax was observed in the SN tissues from PPARαKO sham-operated mice (**C**). MPTP caused an increase in Bax expression in PPARαWT (**D**) and PPARαKO mice. PEA treatment reduced the degree of positive staining for Bax in SN from PPARαWY (**E**). On the contrary, positive staining for Bcl-2 was observed in the brain from PPARαKO sham-operated mice (**F**) while the staining was reduced in MPTP PPARαWT (**G**) and significantly reduced in PPARαKO ice (**H**). PEA treatment attenuated the loss of positive staining for Bcl-2 in SN from MPTP-subjected PPARαWT mice (**I**). The assay was carried out by using Optilab Graftek software on a Macintosh personal computer (CPU G3-266). *p<0.01 vs. Sham. °*p*<0.01 vs *MPTP*+vehicle.

At 8 days after MPTP intoxication, the appearance of the proapoptotic protein, Bax, in brain homogenates was investigated by Western blot analysis (Figure 7A,A1). Bax levels were appreciably increased in the brain from MPTP-treated PPAR-αWT mice (Figure 7, panels A and A1). The absence of PPAR-α gene significantly increased the expression of Bax induced by MPTP. PEA treatment significantly reduced the expression of Bax in PPAR-αWT mice. Minocycline also reduced the increased expression of this pro-apoptotic protein. In PPAR-αKO mice PEA still reduced Bax expression. Moreover, Bcl-2 expression in brain homogenates was also measured by Western blot (Figure 7B and B1). A basal level of Bcl-2 expression was detected in brain from sham PPAR-αWT and PPAR-αKO mice (Figure 7, panels B and B1). Eight days after MPTP intoxication, the Bcl-2 expression was significantly reduced in MPTP-injected mice. The absence of PPAR-α gene significantly increases the loss of Bcl-2 expression induced by MPTP. As shown in Figure 7B,B1, PEA treatment restored Bcl-2 expression in PPAR-αWT and partially in PPAR-αKO.

### Effect of PEA on neurotrophic factors levels after MPTP intoxication

**Figure 8:**
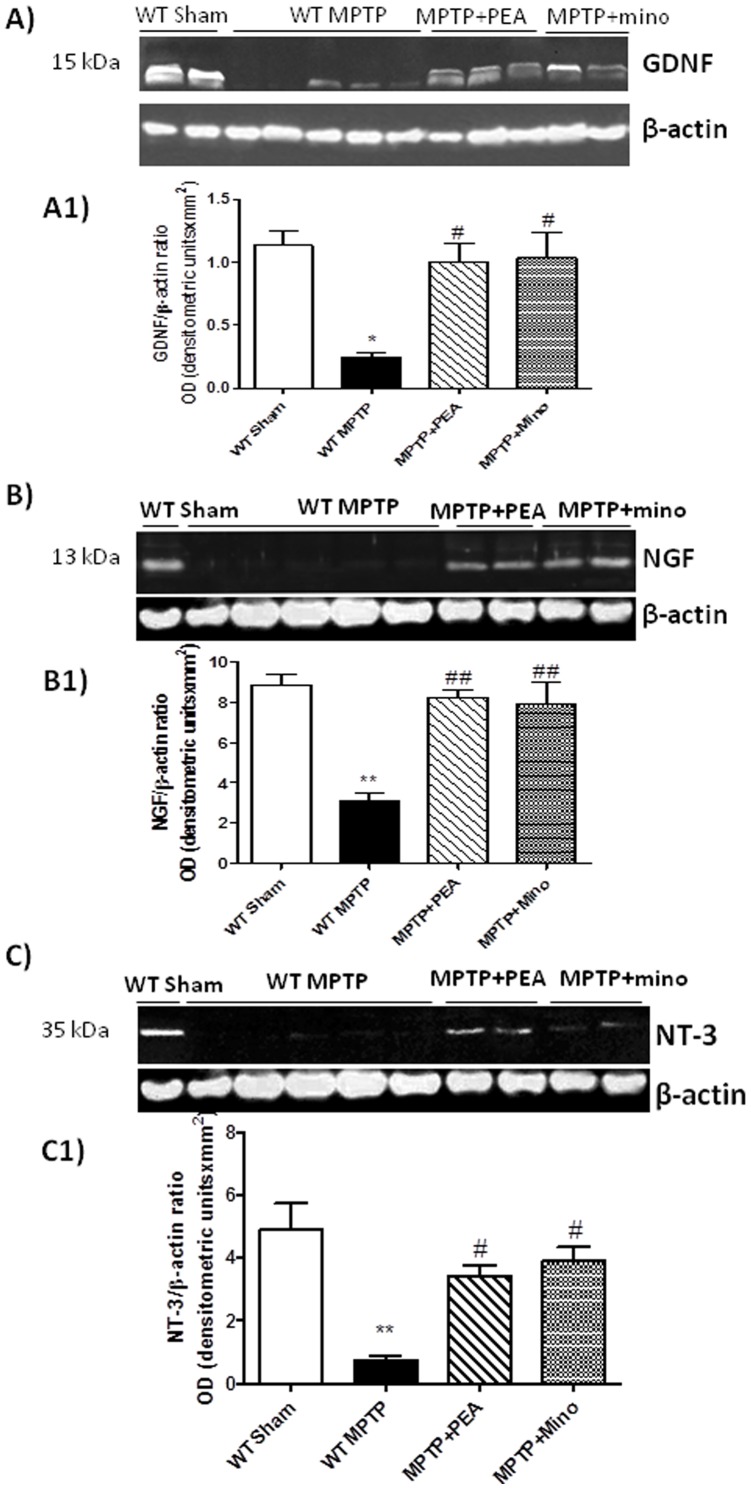
Effect of PEA on GDNF, NGF and NT-3 expression by Western blot analysis. In the brain tissues collected from PPARαWT at 8 days after the MPTP, neurotrophic factors expression was significantly reduced in comparison to sham animals. The treatment with PEA significantly restored in PPARαWT the brain expression of three neurotrophic factors up to that of unreated mice as well as minocycline treatment. *p<0.05 and **p<0.01 vs Sham; #*p*<0.05 and ##*p*<0.05 vs MPTP+vehicle.

To test whether PEA modulates the inflammatory process through regulation of the neutrophic factors levels, we have examined GDNF, NGF and NT-3 levels in the SN by western blot analysis (Figure 8A,B and C, respectively). In the brain tissues collected from PPAR-αWT at 8 days after the MPTP, neurotrophic factors expression levels were significantly reduced in comparison to sham animals. Particularly, in the Western blot analysis it was evident that reduction in GDNF and NGF was about 60% and 63%, whereas for NT-3 the trend of decrease was 75%. The treatment with PEA significantly restore in PPAR-αWT the brain expression of three neurotrophic factors up to that of unreated mice as well as minocycline treatment.

### PEA treatment reduced the histological alteration induced by MPTP administration

**Figure 9:**
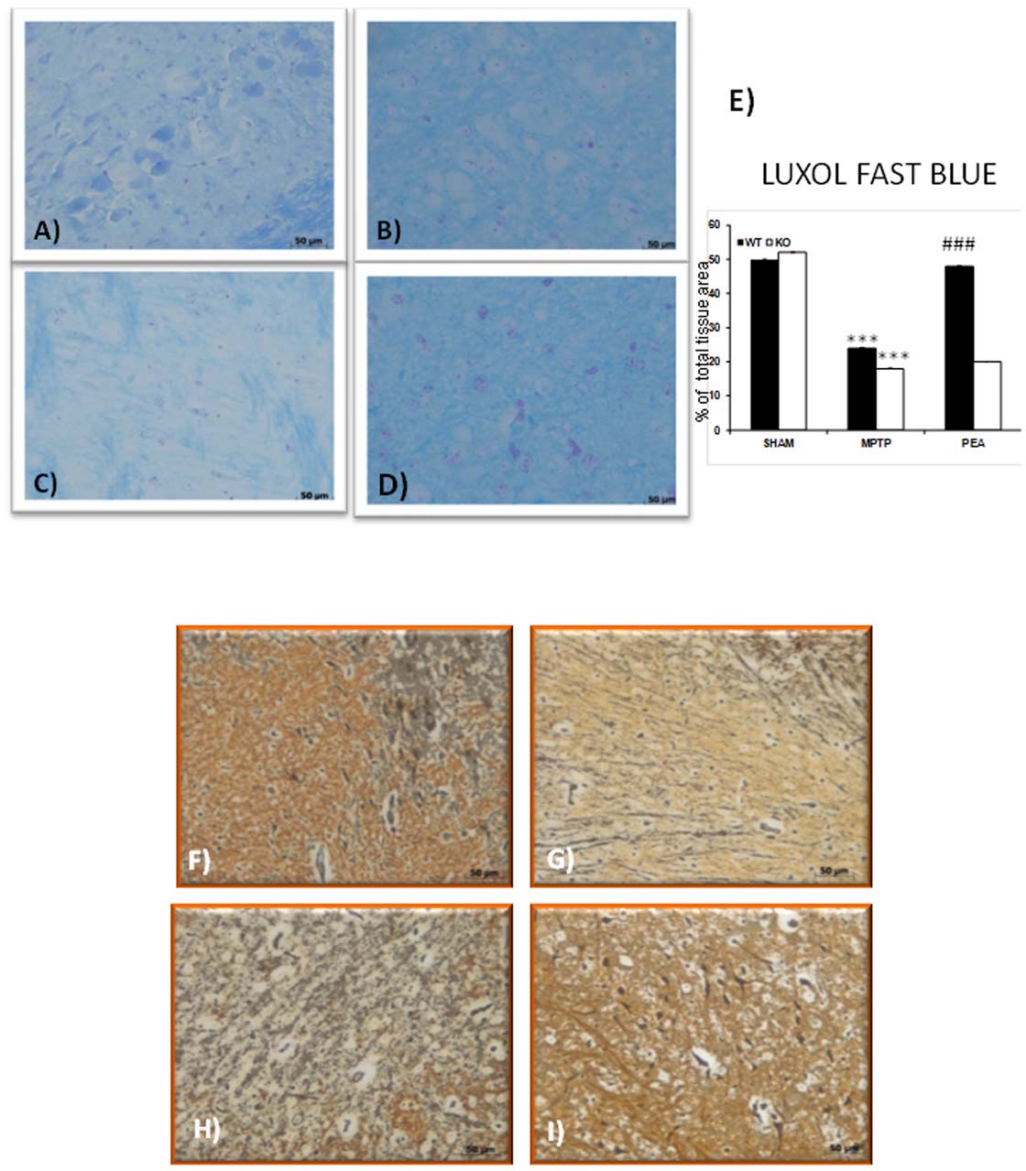
Effect of PEA on myelin structure and presence of granule-laden astrocytes (Gomori stains). In sham PPARαKO animals (A and E), myelin structure was clearly stained by Luxol fast. At 8 days after MPTP injection in PPARαWT mice (B and E), a significant loss of myelin was observed. Myelin degradation was further progressed in PPARαKO mice and myelin negative area was defined in SN (C and E). The treatment with PEA significantly restored in PPARαWT the myelin presence (D and E). In addition, in sham PPARαWT mice (data not shown) or sham PPARαKO mice (F) a significant presence of astrocytes exhibiting an affinity for chrome-alum hematoxylin and aldehyde fuchsin was observed in the SN as well as in the vessels. On the contrary, a significant alteration of the Gomori positive localization was observed in the SN from MPTP-injected PPARαWT mice (G). A more alteration of Gomori positive localization was observed in MPTP injected-PPARαKO mice (H). PEA treatment reduced the alteration of the Gomori positive localization in PPARαWT mice (I).

The severity of the SN alteration was observed at 8 days after MPTP intoxication. Myelin structure was observed by Luxol fast blue staining (Figure 9A,B,C,D,E). In sham PPAR-αWT (data not shown) and PPAR-αKO animals (Figure 9A and E), myelin structure was clearly stained by Luxol fast. At 8 days after MPTP injury in PPAR-αWT mice (Figure 9B and E), a loss of myelin was observed. Myelin degradation was further progressed in PPAR-αKO mice and myelin negative area was clearly defined in the SN (Figure 9C). The treatment with PEA restored in PPAR-αWT (Figure 9D and E) but not in PPAR-αKO the myelin presence (data not shown).

In addition, in sham PPAR-αWT mice (data not shown) or sham PPAR-αKO mice (Figure 9F) a presence of granule-laden astrocytes exhibiting an affinity for chrome-alum hematoxylin and aldehyde fuchsin (Gomori stains) in SN was observed in the SN as well as in the vessels. On the contrary an important alteration of the Gomori positive localization was observed in the SN from MPTP-injected PPAR-αWT mice (Figure 9G). A more alteration of Gomori positive localization was observed in the SN from MPTP injected-PPAR-αKO mice (Figure 9H). PEA treatment reduced the alteration of the Gomori positive localization in PPAR-αWT mice (Figure 9I) but not in PPAR-αKO mice at 8 days after MPTP treatment.

### PEA treatment reduced the activation of astrocytes induced by MPTP injection

**Figure 10:**
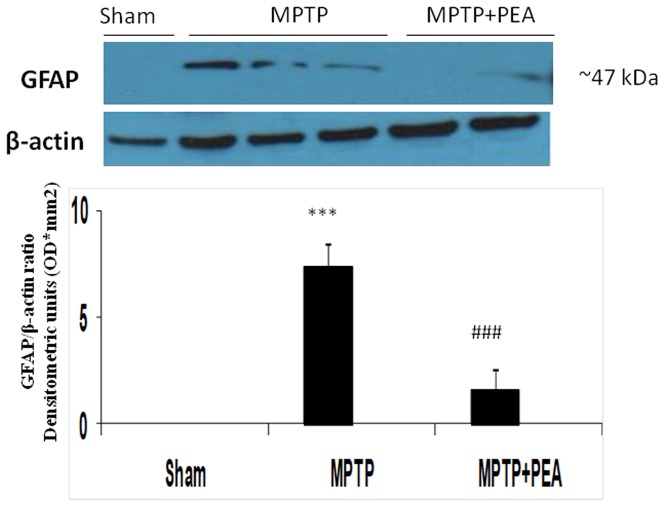
Effect of PEA on GFAP expression, a marker of the activation of astrocytes. Western blot analysis showed a significant expression of GFAP in SN. PEA treatment significantly reduced the activation of astrocytes after MPTP injection. The relative expression of the protein band (∼50 kDa) was standardized for densitometric analysis to β-actin levels, and reported in panel **a1** are expressed as mean ± s.e.m. from n = 5/6 brains for each group. ***P<0.001 *versus* sham, ^###^ *P*<0.001 *versus* MPTP+vehicle.

The results from Western blot analysis demonstrated that PEA attenuated the increase in GFAP, a marker of astrocyte activation, on the SN on day 8 post-MPTP injection (Figure 10)

### Effects of injury and PEA treatment on PPAR-α Expression

**Figure 11:**
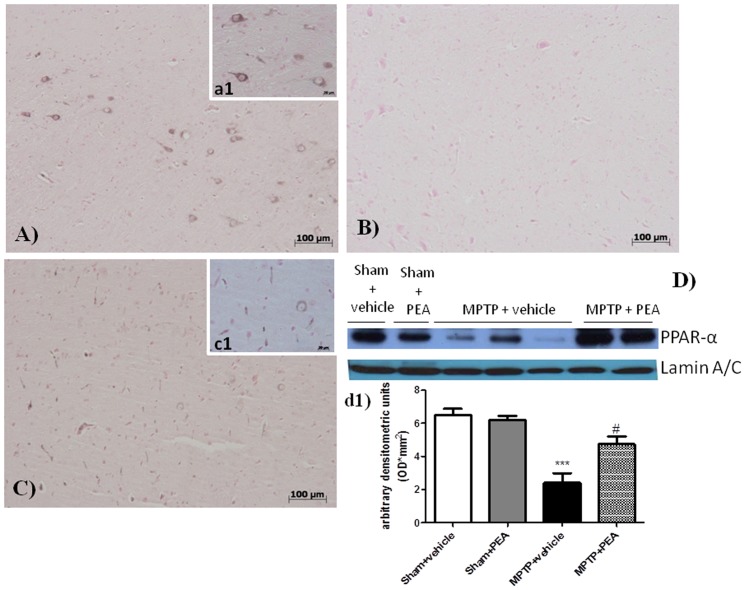
Effect of PEA on PPAR-α expression. Western blot analysis showed a basal expression of PPAR-α in SN. MPTP injection significantly reduced the expression of this specific nuclear receptor expreesion. PEA treatment is able to restore PPAR-α espression. The relative expression of the protein band (∼55 kDa) was standardized for densitometric analysis to lamin levels, and reported in panel **a1** are expressed as mean ± s.e.m. from n = 5/6 brains for each group. ***P<0.001 *versus* sham, ^#^ *P*<0.05 *versus* MPTP+vehicle.

Western blot analysis shows that PPAR-α (∼55 kDa) was expressed in uninjured spinal cords (Figure 11, panel D and d1). Twenty-four hours after SCI, PPAR-α protein expression was significantly reduced in spinal cord homogenates. PEA treatment significantly prevented the SCI-induced down-regulation of PPAR-α.

## Discussion

Recently, the involvement of neuroinflammation and microglial activation in the pathogenesis of PD has been emphasized [44], [45], [46]. Results from neurotoxin models of PD, corroborating findings obtained in transgenic animal models and epidemiological studies, strongly support the hypothesis that this neurodegenerative disease is not purely neuronal, as it has been previously considered [47]. Inflammation in PD is not any longer considered a non-specific consequence of neuronal degeneration as it was originally thought to be. Indeed, neuroinflammation may aggravate the course of the disease and, as has recently been suggested, may be a primary factor in some cases of PD [47], [48].

Unfortunately, current therapies do not address this neuroinflammation problem, being focused on ameliorating the symptoms of dopamine loss rather than on the underlying causes of injury to dopaminergic neurons. To date, all drugs with proven neuroprotective activity in animal models of PD have failed to show significant efficacy in clinical trials. Thus, there is an urgent need for neuroprotective agents that can be administered during the course of the disease to prevent or, at least, limit PD progression. Targeting the signaling pathways in glial cells responsible for neuroinflammation represents a promising new therapeutic approach designed to preserve remaining neurons in PD patients, thereby extending the window of efficacy of existing symptomatic drugs in order to better maintain quality of life.

Thus the study shows that PEA hinders dopaminergic cell death and glial activation in the chronic MPTP model of PD in mice and investigates the molecular mechanisms by which PEA protects mouse nigrostriatal neurons from MPTP-induced neurotoxicity and neuroinflammation.

Several studies suggest that glial cells in neurodegenerative diseases are affected more than neurons by apoptotic cell death [9], [49]. In an effort to prevent or diminish levels of apoptosis, we have demonstrated that the treatment with PEA attenuates the degree of apoptosis. Several studies have postulated that preserving Bax, a pro-apoptotic gene, plays an important role in developmental cell death [50] and in CNS injury [51]. We have identified proapoptotic transcriptional changes, including upregulation of proapoptotic Bax and down regulation of antiapoptotic Bcl-2, by immunohystochemical staining and Western blot analysis. We demonstrated that the treatment with PEA reduced Bax expression, while on the contrary, Bcl-2 expressed much more in mice treated with PEA. On the other hand, in our opinion the observed effects of PEA treatment on apoptosis are at least partially dependent on the activation of PPAR-α. In fact, it has been demonstrated that PPAR-α suppress the apoptosis of hepatocytes [52]. In addition, Inoue and colleagues have clearly demonstrated that apoptosis in human umbilical vein endothelial cells was prevented by transfection with the gene for the human full-length PPAR-α, or acyl-coenzyme A synthetase (AcylCS) [53].

Chronic treatment with PEA protected mouse nigrostriatal neurons from MPTP-induced neurotoxicity/neuroinflammation while ameliorating PD-associated motor deficits. A comparison with minocycline, a semisynthetic tetracycline with beneficial anti-inflammatory and dopaminergic neurons protective activities, was performed. Chronic treatment with PEA (10 mg/kg, i.p.), initiated 24 hr after MPTP injection, protected against MPTP-induced loss of TH+ neurons in the SNc. Treatment with PEA reduced MPTP-induced microglial activation, S100β overexpression, and protected against the alterations of microtubule-associated protein 2a,b (MAP2)-, DAT-, nNOS-positive cells in the SN. Furthermore, chronic PEA reversed MPTP-associated motor deficits, as revealed by the analysis of forepaw step width and percentage of faults. Genetic ablation of PPAR-α in PPAR-αKO mice exacerbated MPTP systemic toxicity, and chronic administration of PEA protected against MPTP-induced neurotoxicity, in part dependently of PPAR-α activation.

It is important to note that PEA failed to rescue behavioral studies and molecular markers induced by MPTP injection in PPAR-α KO mice, such as S-100, MAP-2, PAR expression, and luxol fast blue staining. However, several protective effects of PEA, such as on iNOS, Bax and Bcl-2 expression, are still present in PPAR-αKO mice after treatment, pointing to another targets different from PPAR-α.

To our knowledge, there are no other publications which investigate the effect of PEA on MPTP neurotoxin model. Moreover, this is the first report that documents the molecular mechanisms by which PEA protects mouse nigrostriatal neurons from MPTP-induced neurotoxicity and neuroinflammation. Our results indicate that the PEA is neuroprotective even when administered once the insult has been initiated. This point is of particular importance, as the lack of PD biomarkers and the difficulties of early diagnosis make the pharmacotherapy of PD possible only when dopamine neuronal loss is advanced and the first symptoms have appeared.

PEA, administered once the insult has been initiated, cuts off the link inflammation-gliosis-mitochondrial dysfunction and may be represent a good neurotrophic support. A comparison of PEA against minocycline, also known having neuroprotective activity, demonstrated superior recovery in the PEA-treated animals vs minocycline-treated mice.
